# Reliability of Dr. Leverson’s Vaccination Testimony

**Published:** 1898-08

**Authors:** Montague R. Leverson

**Affiliations:** Fort Hamilton, N. Y.


### RELIABILITY OF DR. LEVERSON'S VACCINATION TESTIMONY.

FORT HAMILTON, N. Y., July 13th, 1898.

EDITOR OF THE HOMŒOPATHIC PHYSICIAN:

As intimated by Dr. C. H. Oakes, of Livermore Falls, in his letter published in your journal for June, the value of the abstract of the Royal British Commission testimony depends upon its reliability.

Your readers will remember that, accepting the criticism of a correspondent who did not quite like the manner in which I was at first presenting the testimony, I have for some time given nearly every statement of importance, certainly everything which could be a subject of question, in the very words in which they are given in the Blue Books, so that it is difficult to see how any room remains for questioning the accuracy of what is now being published in THE HOMŒOPATHIC PHYSICIAN. Appreciating fully the truth of Dr. Oakes' position, *I wish to have further security for the accuracy of the abstracts.* I therefore place at the free disposal of any person who will come to my house for the purpose, all the Blue Books for comparison with the abstracts. Further, I suggest the appointment of a committee to verify the same.

Perhaps some old school doctor could be induced to ask the Academy of Medicine to appoint such committee, and one of the homœopathic bodies might also do the like. By this means an authority could be given to the abstracts which would forever silence all possibility of cavil.

Yours very truly,

MONTAGUE R. LEVERSON.

